# Phage-display reveals interaction of lipocalin allergen Can f 1 with a peptide resembling the antigen binding region of a human γδT-cell receptor

**DOI:** 10.1515/hsz-2020-0185

**Published:** 2020-09-16

**Authors:** Matthias Habeler, Bernhard Redl

**Affiliations:** Institute of Molecular Biology, Medical University Innsbruck, Innrain 80, A-6020 Innsbruck, Austria

**Keywords:** binding studies, bio-panning, high-throughput assay, immunogenicity, T-cells.

## Abstract

Although some progress has been achieved in understanding certain aspects of the allergenic mechanism of animal lipocalins, they still remain largely enigmatic. One possibility to unravel this property is to investigate their interaction with components of the immune system. Since these components are highly complex we intended to use a high-throughput technology for this purpose. Therefore, we used phage-display of a random peptide library for panning against the dog allergen Can f 1. By this method we identified a Can f 1 binding peptide corresponding to the antigen-binding site of a putative γδT-cell receptor. Additional biochemical investigations confirmed this interaction.

Many major allergens from domestic farm and pet animals like cows, horses, dogs, cats or rodents belong to the lipocalin superfamily (Hentges et al. 2014; Hilger et al. 2012; Virtanen et al. 2012). Immunologically, lipocalin allergens have been hypothesized to reside on the border between self and foreign (Nilsson et al. 2014; Virtanen et al. 2012) as several mammalian lipocalins exhibit sequence homologies between 40 and 60% over species. Therefore, they are only weakly recognized by the adaptive immune system due to the absence of high-avidity reactive T-cells following negative thymic selection. In turn, weak T-cell reactivity may favour a T_H_2-dominated immune response resulting in allergy development. However, although there are some new findings concerning the molecular mechanism of lipocalin allergenicity (Hufnagel et al. 2018; Klaver et al. 2019), many aspects still remain unclear. Therefore, investigations concerning the interaction of lipocalins with components of the human immune system may be helpful to elucidate their allergenic mechanism. Our idea was to use a more global approach for such an interaction screening. For this purpose, we used a phage-display based method, which was in general described to be useful in imitating the humoral immune response (Skerra 2003). As bait, we used a recombinant dog allergen Can f 1, a salivary protein present in dog hairs and dander (Konieczny et al. 1997), which is homologous to human tear lipocalin (Glasgow et al. 2002; Redl 2000). Since it is almost impossible to have all the components of the immune system represented by a phage library, a random 12-mer peptide library was used for screening. Peptide phage libraries have successfully been used in biomedical science (Wu et al. 2016). The library used in this study (NEB Ph.D.12), consists of a linear dodecapeptide and short linker sequence fused to the N-terminus of pIII of M13 phage. It contains a complexity of 10^9^ independent clones, which represents only a tiny fraction of the 4.1 × 10^15^ possible 12-mer peptides, however. Nevertheless, this type of peptide library has proven to be of sufficient complexity to yield several DNA sequences encoding the same peptide motif. Interestingly, when panned against Taxol, the Ph.D.-12 library successfully identified peptides corresponding to the binding site of its natural ligand Bcl-2 (Rodi et al. 1999). This demonstrated the applicability of unstructured peptides to mimic three-dimensional ligand binding sites, which we sought to exploit for the identification of novel Can f 1 interacting proteins.

We performed three rounds of biopanning for Can f 1-binding peptides and then isolated and sequenced the DNA of 10 different phages. Selection against Can f 1 resulted in the identification of two different peptides with the vast majority (9 out of 10) carrying the sequence KLWSIPTNFLLP, while NAKYLPTILGRL was only found once. We next subjected the major peptide KLWSIPTNFLLP to local alignment search (BLAST) against the *Homo sapiens* protein database to identify putative interacting proteins featuring these or highly similar amino acid sequences. The top 10 hits are outlined in Table 1. KLWSIPTNFLLP was most similar to the antigen-binding CDR3 of a γδT-cell receptor isolated from human nasal mucosa (Hirata et al. 2000). Lower similarity was found to sequences from oligodendrocyte-myelin glycoprotein, a plasma membrane protein involved in neuron myelination, and enzymes of the phosphoinositide cascade.

**Table 1:** List of top 10 BLAST-hits for Can f 1 binding peptide KLWSIPTNFLLP after searching against *Homo sapiens* database (Blastp NCBI).

#	Protein	Aligned sequence	*E*-value	Accession number	Localisation
1	T-cell receptor delta chain Vd1	WGVPSNFLL	2.9	BAB16938.1	Plasma membrane
2	hCG2045700	LWSRVIPLKGNFLLP	3	EAX01944.1	
3	Phosphatidylinositol 4,5-bisphosphate 3-kinase catalytic subunit beta isoform isoform 1	SIPVDFLLP	4.1	NP_006210.1	Nucleus
4	Phosphoinositide-3-kinase, catalytic, beta polypeptide, isoform CRA_b	SIPVDFLLP	4.1	EAW79054.1	Nucleus, cytosol
5	Oligodendrocyte-myelin glycoprotein	KLWTVPTN	4.1	AAA59970.1	Plasma membrane
6	Oligodendrocyte myelin glycoprotein variant, partial	KLWTVPTN	4.1	BAD96383.1	Plasma membrane
7	Oligodendrocyte-myelin glycoprotein precursor	KLWTVPTN	4.1	NP_002535.3	Plasma membrane
8	Oligodendrocyte-myelin glycoprotein	KLWTVPTN	4.1	CAA35991.1	Plasma membrane
9	Phosphoinositide-3-kinase, catalytic, beta polypeptide, isoform CRA_c	SIPVDFLLP	4.1	EAW79056.1	Plasma membrane
10	hCG2042551, partial	IPYNFLLP	12	EAW96483.1	

Protein name, aligned peptide sequence, expect value, NCBI accession number and subcellular localisation are indicated.

Furthermore, we applied peptide secondary structure prediction by PEP-FOLD 3 (Shen et al. 2014) to model the structure of this peptide (Figure 1). KLWSIPTNFLLP is likely to adopt a loop structure around P_6_ which is stabilized by internal peptide backbone hydrogen bonding. Its small hydrophilic core-T_7_N_8_ is flanked by bulky hydrophobic side chains while charged groups are only found at the peptide termini. Its loop configuration may reflect the natural structure of the antigen-binding site in γδT-cell receptors (TCRs). Its core section -W_3_S_4_I_5_P_6_T_7_N_8_F_9_L_10_L_11_- is almost identical to the CDR3 peptide’s, -WGVPSNFLL-, safe for the functionally conservative mutations I_5_→V and T_7_→S and the substitution of S_4_ by G. From a biochemical standpoint, both these peptides may behave very similarly. Therefore, we further investigated their interaction with Can f 1.

**Figure 1:**
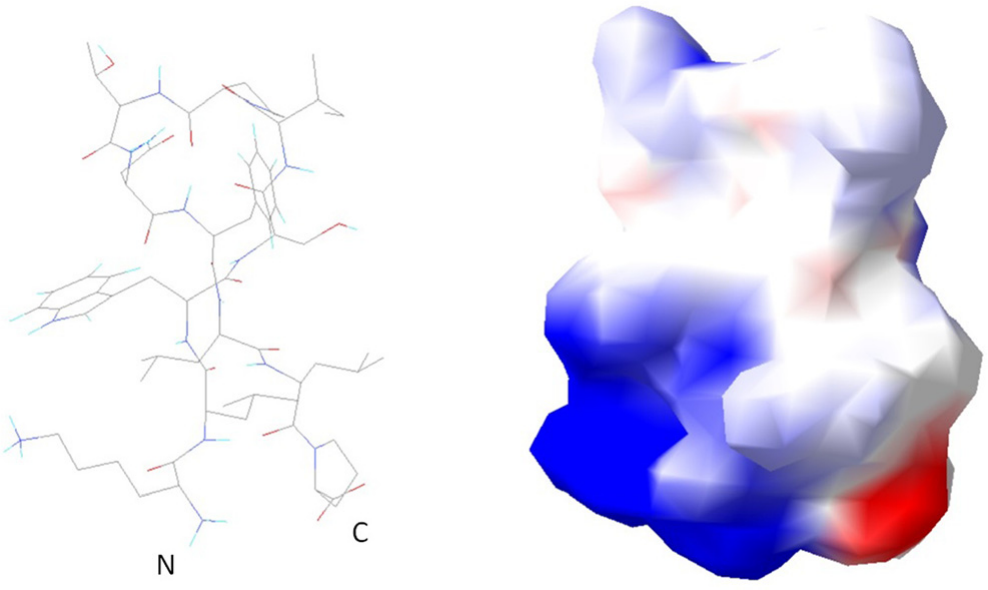
Secondary structure prediction (by PEP-FOLD 3): stick model and electrostatic surface potential for Can f 1-binding peptide KLWSIPTNFLLP, derived from phage-display. Amino-(N) and carboxy-termini (C) are indicated.

Due to its hydrophobic properties, we hypothesized binding of KLWSIPTNFLLP into Can f 1’s ligand cavity. Therefore, ANS (8-anilinonaphthalene-1-sulfonic acid) binding and displacement assays were used to study this supposed mode of interaction. The fluorescent probe ANS readily bound into the hydrophobic binding cavity inside Can f 1’s β-barrel. An increase of specific fluorescence signal at 460 nm by more than 20-fold over free ANS signal was observed upon interaction (Figure 2A). A *K*_*d*_ of 2.1 μM for ANS binding to Can f 1 was calculated, which is well within the range of lipocalin-ligand affinities previously described (Breustedt et al., 2006). Therefore, the Can f 1-ANS complex was suitable for studying interaction properties between Can f 1 and the phage display-derived peptide KLWSIPTNFLLP. For comparison, we tested the displacement of ANS from Can f 1 by increasing concentrations of palmitic acid, a typical ligand of the human Can f 1 homologue Lcn1 (Gasymov et al. 1999), which also binds to several other lipocalins (Akerstrom et al. 2000). Indeed, palmitic acid progressively replaced ANS in the binding pocket of Can f 1 (Figure 2B). The obtained ANS displacement model served as a reference to compare the influence of KLWSIPTNFLLP-peptide on the Can f 1-ANS complex. We then tested the impact of the peptide on the fluorescence of the Can f 1-ANS complex. Surprisingly, this assay revealed a peptide concentration-dependant increase in fluorescence instead of a decrease, which is typically observed for other lipocalin ligands such as palmitic acid (Figure 2B). Intriguingly, this phenomenon was only detected when all three components–Can f 1, ANS and peptide–were present in the same sample, while peptide and ANS alone did not produce a fluorescent signal. We included two additional controls, the originally published CDR3 peptide GSWGVPSNFLLI and a scrambled control peptide (FLSPLKILTWNP, identical aa composition as the phage display peptide, but in different order), in our experiment. As depicted in Figure 2B, the CDR3 peptide produced a very similar effect as the phage display derived peptide, while the scrambled control peptide displayed no change in signal.

**Figure 2:**
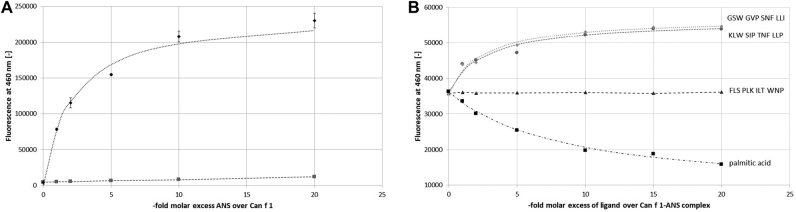
Fluorescence-based binding studies. (A) Binding of ANS to Can f 1 (black). 1 μM delipidated Can f 1 (20 μg/ml) were mixed with a range of ANS concentrations. Samples (100 μl per well) were prepared in triplicates and transferred to black half area 96-well microplates (Greiner Bio-One, Rainbach, Austria). Fluorescence measurements were performed on a Fluostar OMEGA plate reader (BMG Labtech, Ortenberg, Germany) with OMEGA and MARS software. Can f 1-ANS complexes were excited at 355 nm and their fluorescence recorded at 460 nm. Can f 1 binds ANS with *K*_*d*_ = 2.1 µM. ANS baseline (grey). Further experimental details are given in Supplementary material. (B) Effect of palmitic acid and different peptides on the Can f 1-ANS complex. Can f 1-ANS was titrated with increasing concentration of palmitic acid (■). Palmitic acid binds into and displaces ANS from Can f 1’s ligand binding pocket. The Can f 1-ANS complex was also incubated with increasing amounts of the phage display-derived peptide KLW SIP TNF LLP (●) and the δ TCR CDR3-peptide GSW GVP SNF LLI (◆). These peptides did not displace ANS from to the Can f 1-ANS complex but increased the fluorescence in a dose dependant manner. No effect of a scrambled control peptide (FLSPLKILTWNP) on the Can f 1ANS complex was observed (▲). Measurements were done as described for panel (A). Data points represent the mean of duplicates. Experimental details are given in online Supplementary material.

These data indicate an interaction between peptide and Can f 1-ANS complex in which binding occurs without the displacement of ANS. Instead, presence of the peptide appears to change the chemical environment near the ANS molecule inside Can f 1’s cavity, thereby altering the fluorescent properties of the complex. Two potential modes of interaction capable of triggering this shift include: First, a shallow binding of the peptide toward the open end of the β-barrel, which might allow for penetration of the peptide’s hydrophobic side chains into the binding pocket where they contact the ANS molecule. Or second, binding to a remote site on the surface of Can f 1, which induces a conformational change affecting the ligand binding cavity.

In order to confirm the interaction of Can f 1 and the originally published δ TCR CDR3-peptide, we performed another orthogonal binding assay. For this purpose, we fused the peptide sequence SWGVPSNFLLIVSDKLIFG (antigen-binding site in bold letters while the rear sequence is part of the TCR backbone) to the C-terminus of the SH2 domain of human intracellular phosphatase SHP-2 for expression as a fusion protein from *E. coli* (Supplementary Figure S1). This approach was chosen to avoid solubility problems due to the peptide’s hydrophobic nature (Fairlie et al. 2002). The resulting fusion gene coded for a mature protein featuring an N-terminal Strep-tag, the fusion partner SH2 domain followed by the δ TCR CDR3- peptide and a C-terminal His-tag (Supplementary Figure S2).

We then used an enzyme-linked assay to study binding of the recombinant δ TCR CDR3-peptide to Can f 1. Microplates coated with Can f 1 were incubated with SWGVPSNFLL-SH2-fusion protein. The N-terminal Strep-tag was used for detection and quantification of captured fusion protein using StrepTactin-HRP.

It is evident from Figure 3 that increased binding of SH2-CDR3 occurred in wells coated with Can f 1 in comparison to only BSA-blocked control wells. Moreover, this binding was markedly reduced by competition with a 10-fold molar excess of phage display-peptide KLWSIPTNFLLP, indicating that binding of SH2-CDR3 to Can f 1 is indeed mediated by the CDR3 peptide. Therefore, it is reasonable to assume that the δ TCR CDR3-peptide SWGVPSNFLL may also mediate the recognition of allergenic Can f 1 protein by γδT-cells *in vivo*.

**Figure 3:**
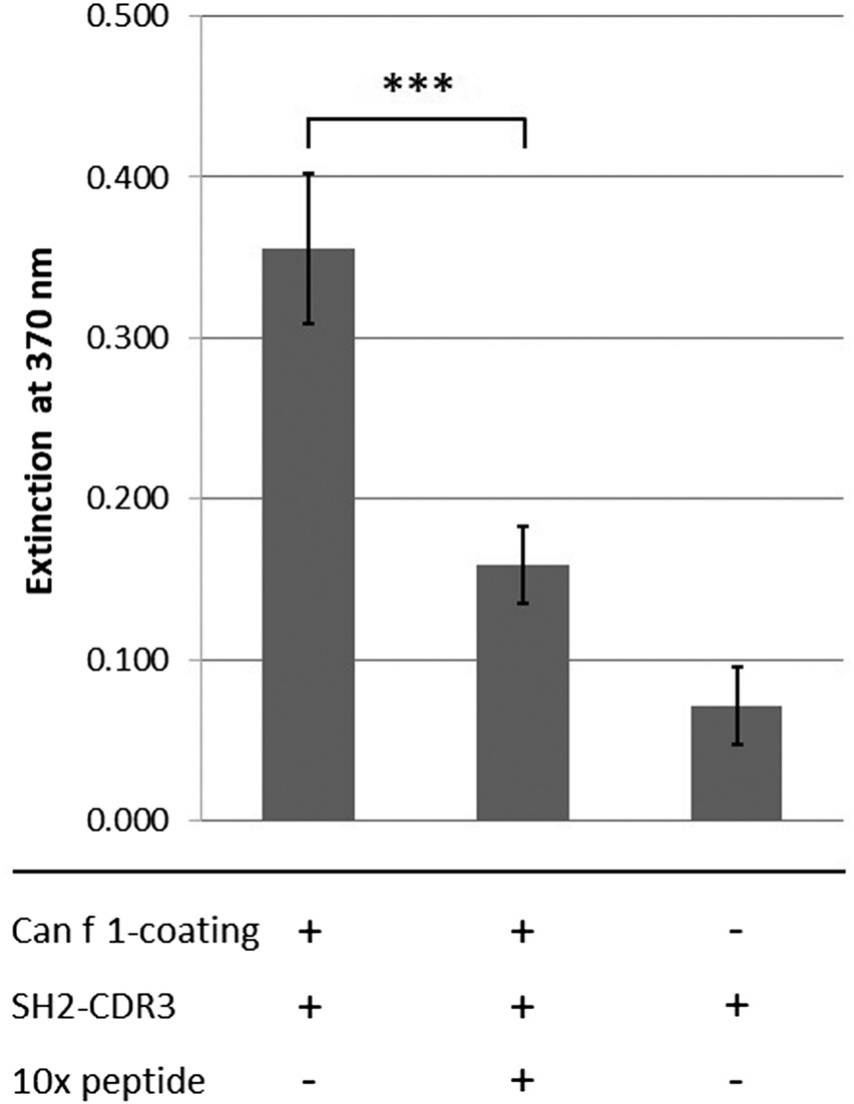
SH2-CDR3 fusion protein binds specifically to Can f 1-coated wells. 96-well plates were coated with Can f 1 protein and/or blocked with BSA only. SH2-CDR3 fusion protein featuring the sequence-AWGVPSNFLL-was added in the absence or presence of a 10-fold molar excess of phage display-derived peptide KLWSIPTNFLLP. Captured protein was measured using StrepTactin-horseradish peroxidase conjugate (SH2-CDR3 being Strep-tagged) and tetramethylbenzidine substrate. SH2-CDR3 fusion protein showed increased binding to Can f 1-coated wells compared to BSA-blocking only. This interaction was significantly inhibited (*p* = 0.0038) in the presence of KLWSIPTNFLLP-peptide. Experiments were done in triplicates. Detailed information is given in the online Supplementary material.

Our study supports previous indications for a role of γδT-cells in allergenicity as discussed below. In general, γδT-cells are a minor population of lymphocytes expressing the γδT-cell receptor, which constitute about 5% of T-cells in peripheral blood but as much as 50% in epithelial and mucosal tissues, where they may play an important immunological role. In contrast to αβT-cells, which canonically require antigen processing and presentation by professional antigen-presenting cells (APCs), γδT-cells are capable of directly recognizing unprocessed antigens. In fact, Rock et al. (1994) suggested ligand binding by γδT-cells to be more akin to immunoglobulins (Igs) than αβT-cell receptors (TCRs). Much like in immunoglobulins, where the heavy chain’s complementary-determining region 3 (CDR3) contributes greatly to receptor diversity and represents the principal antigen binding site, the TCR δ CDR3 has been proposed as the major ligand interaction site. Since then, this assumption has been proven valid e.g. by the work of Chen et al. (2008), who successfully applied Vδ2 CDR3-derived peptides from ovarian epithelial carcinoma-infiltrating T lymphocytes for the enrichment of corresponding antigen sequences from the same Ph.D. random 12-mer peptide library applied in our study. Although only a small number of proteinaceous antigens for γδ TCRs have been identified to date, it has been argued that proteins might represent the bulk of antigens recognized by γδ TCRs (Born et al. 2011). Indeed, already in 1995, a study on house dust-mite (*Dermatophagoides pteronyssinus*)-allergic asthmatics (Spinozzi et al. 1995) found a pulmonary population of Der p 1 antigen-specific γδT-cells. Upon Der p 1-stimulation these cells proliferated and secreted interleukins (ILs) 4 and 5, thereby recruiting further specialized immune cells and mounting a T_H_2-type allergic response. However, the role of γδT-cells in allergic diseases is anything but clearly defined (Pawankar 2000; Zheng and Yang 2014). Although they seem to be required to elicit allergic airway inflammation in immunized, ovalbumin-challenged mice, where they produce T_H_2-type cytokines (Zuany-Amorim et al. 1998), γδT-cells have also been reported to fulfil a regulatory role by suppressing T_H_2 acute airway inflammation and constraining airway remodelling (Murdoch et al. 2014). The identification of a γδ TCR CDR3-derived peptide as a potential Can f 1-interacting molecule is a novel and intriguing finding and may induce further research to enlighten the the role of these cells in lipocalin allergenicity. Thus, additional cellular studies have to be performed to proof these results *in vivo*.

## Supplementary Material

Supplementary Material
