# Supplementary material for: Phage-display reveals interaction of lipocalin allergen Can f 1 with a peptide resembling the antigen binding region of a human γδT-cell receptor
Source: Biol Chem. 2020 Sep 16;402(4):433–7. doi: 10.1515/hsz-2020-0185 (PMC10883907; doi:10.1515/hsz-2020-0185)
Supplement: Supplementary file 1 — Supplementary Material [file j_hsz-2020-0185_suppl.pdf]

Phage-display reveals interaction of lipocalin allergen Can f 1 with a peptide resembling the antigen binding region of a human γδT- cell receptor

Matthias Habeler and Bernhard Redl

Supplementary Material

Supplementary Table S1:

#	DNA insert	Peptide
**1**	TCTCACTCT**AAGCTTTGGTCGATTCCGACTAATTTTTTGTTGCCT**GGTGGAGGTTCGGCCGAA	KLWSIPTNFLLP
**2**	TCTCACTCT**AATGCGAAGTATCTTCCGACTATCCTGGGTCGCCTG**GTGGAGGTTCGGCCGAA	NAKYLPTILGRL
**3**	TCTCACTCT**AAGCTTTGGTCGATTCCGACTAATTTTTTGTTGCCT**GGTGGAGGTTCGGCCGAA	KLWSIPTNFLLP
**4**	TCTCACTCT**AAGCTTTGGTCGATTCCGACTAATTTTTTGTTGCCT**GGTGGAGGTTCGGCCGAA	KLWSIPTNFLLP
**5**	TCTCACTCT**AAGCTTTGGTCGATTCCGACTAATTTTTTGTTGCCT**GGTGGAGGTTCGGCCGAA	KLWSIPTNFLLP
**6**	TCTCACTCT**AAGCTTTGGTCGATTCCGACTAATTTTTTGTTGCCT**GGTGGAGGTTCGGCCGAA	KLWSIPTNFLLP
**7**	TCTCACTCT**AAGCTTTGGTCGATTCCGACTAATTTTTTGTTGCCT**GGTGGAGGTTCGGCCGAA	KLWSIPTNFLLP
**8**	TCTCACTCT**AAGCTTTGGTCGATTCCGACTAATTTTTTGTTGCCT**GGTGGAGGTTCGGCCGAA	KLWSIPTNFLLP
**9**	TCTCACTCT**AAGCTTTGGTCGATTCCGACTAATTTTTTGTTGCCT**GGTGGAGGTTCGGCCGAA	KLWSIPTNFLLP
**10**	TCTCACTCT**AAGCTTTGGTCGATTCCGACTAATTTTTTGTTGCCT**GGTGGAGGTTCGGCCGAA	KLWSIPTNFLLP

DNA sequences and deduced amino acid sequences of 10 Can f 1 binding phages after three rounds of panning. Peptide-coding DNA sequences are in bold letters.

Supplementary Figure S1:

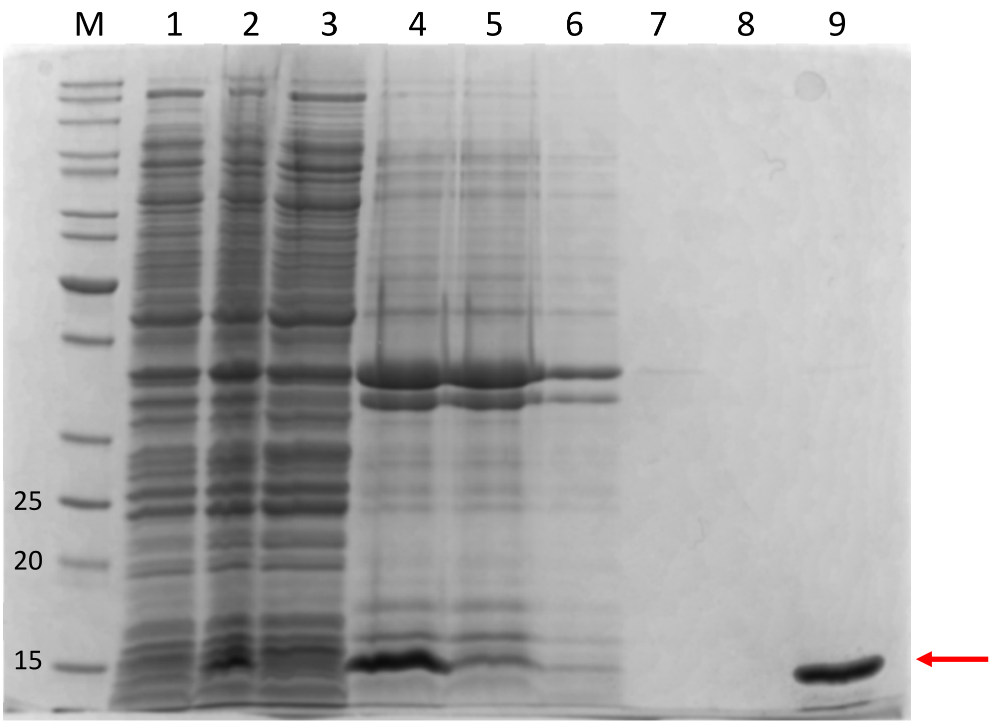

Purification of Strep-SH2-CDR3-His fusion protein from *E. coli* inclusion bodies. Non-induced (1) and induced (2) sample taken during expression. Recombinant protein is absent in the soluble fraction (3) and was extracted from insoluble inclusion bodies (4). The solubilized protein was captured by Ni-NTA chromatography: Flow-through after binding (5) and washing/on column-refolding steps (washes with 4, 2 and 0.25 M urea correspond to lanes 6, 7 and 8). Purified and refolded protein (expected MW 15.9 kDa) eluted from the column (9, indicated by arrow). Molecular weight marker (M).

Supplementary Figure S2:

```
                                                              XhoI
101  CTCAGCTTCC TTTCGGGCTT TGTTAGCAGC CGGATCTCAG TGGTGGTGGT GGTGGTGCTC GAGGCCGAAA ATCAGTTTGT CGCTCACGAT CAGCAGAAAG
     GAGTCGAAGG AAAGCCCGAA ACAATCGTCG GCCTAGAGTC ACCACCACCA CCACCACGAG CTCCGGCTTT TAGTCAAACA GCGAGTGCTA GTCGTCTTTC
 -2                                          ••• His His His His His His Glu Leu Gly Phe Ile Leu Lys Asp Ser Val Ile Leu Leu Phe Asn·

                          EcoRI
                               BamHI
201  TTAGACGGCA CACCCCACGC GAATTCGGAT CCGTTCAGAG GATATTTAAG CTCAATGACA TCTCCATTCT TCTCTTTTAA TTGCCCGTGA TGTTCCATGT
     AATCTGCCGT GTGGGGTGCG CTTAAGCCTA GGCAAGTCTC CTATAAATTC GAGTTACTGT AGAGGTAAGA AGAGAAAATT AACGGGCACT ACAAGGTACA
 -2  ·Asn Ser Pro Val Gly Trp Ala Phe Glu Ser Gly Asn Leu Pro Tyr Lys Leu Glu Ile Val Asp Gly Asn Lys Glu Lys Leu Gln Gly His His Glu Met Tyr·

301  AATACTGGAC CAACTCAGCC AAAGTGGCAA ATTTCTCCCC TCCATACAGG TCATAGTAAT CACCAGTGTT CTGAATCTTG ATGTGGGTGA CAGCTCCATT
     TTATGACCTG GTTGAGTCGG TTTCACCGTT TAAAGAGGGG AGGTATGTCC AGTATCATTA GTGGTCACAA GACTTAGAAC TACACCCACT GTCGAGGTAA
 -2  ·Tyr Tyr Gln Val Leu Glu Ala Leu Thr Ala Phe Lys Glu Gly Gly Tyr Leu Asp Tyr Tyr Asp Gly Thr Asn Gln Ile Lys Ile His Thr Val Ala Gly Asn

401  TCTTCTAACG GAAAGTGTGA AGTCTCCAGG GTTACTTTTA CTAGGCCTTG CCAAAAAACT GCCATCAACT CCTCTTGTCA ACAGTAGGTT TTCTGCCTCC
     AGAAGATTGC CTTTCACACT TCAGAGGTCC CAATGAAAAT GATCCGGAAC GGTTTTTTGA CGGTAGTTGA GGAGAACAGT TGTCATCCAA AAGACGGAGG
 -2  Arg Arg Val Ser Leu Thr Phe Asp Gly Pro Asn Ser Lys Ser Pro Arg Ala Leu Phe Ser Gly Asp Val Gly Arg Thr Leu Leu Leu Asn Glu Ala Glu Val·

                                                                   NcoI
501  ACACCAGTGA TATTTGGGTG AAACCATCTC CGCGATGTTT TTTCAAACTG CGGATGGCTC CAAGCCATGG TATATCTCCT TCTTAAAGTT AAACAAAATT
     TGTGGTCACT ATAAACCCAC TTTGGTAGAG GCGCTACAAA AAAGTTTGAC GCCTACCGAG GTTCGGTACC ATATAGAGGA AGAATTTCAA TTTGTTTTAA
 -2  ·Val Gly Thr Ile Asn Pro His Phe Trp Arg Arg Ser Thr Lys Glu Phe Gln Pro His Ser Trp Ala Met

       XbaI
601  ATTTCTAGAG GGGAATTGTT ATCCGCTCAC AATTCCCCTA TAGTGAGTCG TATTAATTTC GCGGGATCGA GATCTCGATC CTCTACGCCG GACGCATCGT
     TAAAGATCTC CCCTTAACAA TAGGCGAGTG TTAAGGGGAT ATCACTCAGC ATAATTAAAG CGCCCTAGCT CTAGAGCTAG GAGATGCGGC CTGCGTAGCA
```

DNA sequence of the SH2-CDR3 fusion protein gene in pET21d+ backbone (encoded by reverse strand). The CDR3 peptide is given in aa position 11 to 22.

Specific materials and methods

1. Production of recombinant Can f 1.

A codon optimized Can f 1 was synthesized (GenScript, Piscataway, USA), cloned into Qiagen´s pQE70 expression plasmid, produced and purified in M15 *E.coli* according to the manufacturer´s instruction (QIAexpressionist).

2. Phage display:

Library: Ph.D.-12 Phage Display Peptide Library Kit E8110S (New England Biolabs GmbH, Frankfurt, Deutschland)

Materials, general handling of phages and sequencing of inserts was done as described in the Instruction Manual Ph.D Phage Display Libraries from NEB (NEB, Ipswich, USA)

Biopanning for the enrichment of lipocalin-interacting phages: Target rCan f 1 was diluted to 100 µg/mL in coating buffer (100 mM $NaHCO_3$ pH 8.6). 150 µL of this solution was used to coat a single well (per target protein) of a 96-wells microtiter plate (Nunc Maxisorb, Thermo Scientific, Vienna, Austria) overnight. The next morning, the coating solution was discarded and replaced by blocking buffer (5 mg/mL BSA in coating buffer). The microtiter plate was blocked for 60 min at 4 °C. Meanwhile, a 100-fold representation of the original Ph.D.-12 library was prepared by diluting 10 µL (corresponding to 10^{11} pfu equalling 100 times the library complexity of 10^9 individual clones) in 90 µL of TBS-T (TBS + 0.1 % Tween 20). Next, the blocking solution was discarded and the microtiter plate rapidly washed 6 times in 200 µL TBS-T buffer. The prepared phage library was added to the plate and incubated on a rotating shaker at room temperature (RT) for 60 min. Then, the phage suspension was discarded and the plate washed again 10 times in 200 µL TBS-T to remove non-binders. Finally, bound phages were eluted competitively in 100 µL solution of the respective free target (100 µg/mL Can f1 in TBS) on a rotating shaker at RT for 60 min. Panning procedure was repeated three times.

3. Binding studies

3.1. Can f 1-ANS binding studies

1 µM delipidated Can f 1 (20 µg/mL) were mixed with a range of ANS concentrations (0, 1, 2, 5, 10 and 20 µM or -fold molar excess respectively) in 20 mM potassium phosphate buffer pH 7.2 containing 0.86 % acetonitrile at RT. *Acetonitrile was later used as a solvent for the synthetic peptides* and was therefore already included in this experiment to rule out any adverse effects. Samples (100 µL per well) were prepared in triplicates and transferred to black half area 96-wells microplates (Greiner Bio-One, Rainbach, Austria). After 10 min incubation in the dark, fluorescence measurements were performed on a Fluostar OMEGA plate reader (BMG Labtech, Ortenberg, Germany) with OMEGA and MARS software. Can f 1- ANS complexes were excited at 355 nm and their fluorescence recorded at 460 nm. A second series of measurements omitting Can f 1 was performed under identical conditions to establish ANS background signal.

3.2. ANS displacement from Can f 1 by palmitic acid

ANS was displaced from its complex with Can f 1 (both at 1 μM) by increasing concentrations of the hydrophobic ligand palmitic acid. A series of 50-fold palmitic acid stock solutions in EtOH was prepared – 1000, 750, 500, 250, 100 and 50 μM as well as an EtOH blank. The reaction mixture contained 4 μL 500 μg/mL delipidated Can f 1, 1 μL 100 μM ANS, 20 mM potassium phosphate pH 7.2 (see above), 2 μL of respective 50-fold palmitic acid stock and ddH_2O to 100 μL. Measurements were performed as described above.

3.3. Influence of peptides on ANS binding to Can f 1

5 μg of highly pure synthetic peptides, KLWSIPTNFLLP (ChinaPeptides, Shanghai, China), scambled control peptide FLSPLKILTWNP (ProteoGenix, Schiltingheim, France) and peptide GSWGVPSNFLLI (ProteoGenix, Schiltingheim, France) were dissolved in 30 % acetonitrile in ddH2O at a concentration of 700 μM. From this stock solution, a dilution series of 525, 350, 175, 70 and 35 μM in the same solvent was made. The reaction mixture for the ANS assay contained 4 μl 500 μg/mL delipidated Can f 1, 1 μl 100 μM ANS in ddH2O, 20 mM potassium phosphate pH 7.2, 2,85 μL of the corresponding peptide solution, ddH_2O to 100 μl. The final peptide concentrations were 0, 1, 2, 5, 10, 15, 20 μM. Assays were performed as described above.

4.1. Synthesis and cloning of δ TCR CDR3 peptide and cloning into SH2-fusion protein plasmid

Synthesis of the gene encoding the δ TCR CDR3-peptide SWGVPSNFLLIVSDKLIFG was performed by PCR using two codon-optimized overlapping primers, (forward: 5'-ctGGATCCatgggcagctggggtgtgccgtctaactttctgctgatcg**tgagcgacaaactgattttc**-3'and revers: 5'-cgCTCGAGtcattatttttcaaactgcggatggctccacgcagagcc**gaaaatcagtttgtcgctca**-3'), which was then cloned into pASK75 adding a C-terminal Strep-tag onto the construct. It was afterwards subcloned into a SH2 domain containing pET21d+ plasmid. The resulting gene encoded a mature protein combined of an N-terminal Strep-tag, the SH2-fusion partner, the CDR3-peptide (slightly modified to accommodate an additional EcoRI site for control digestion) and a C-terminal His-tag (Supplementary Figure S2).

4.2. Expression, purification of SH2-CDR3 fusion peptide under denaturing conditions and renaturation

A single colony of Rosetta(DE3) cells harbouring pET21d+ SH2-CDR3 plasmid was used to inoculate 12 mL of LB medium (containing 100 μg/mL ampicillin and 25 μg/mL chloramphenicol). The culture was grown overnight at 37 °C on a rotating shaker (x180 rpm). The next morning, 10 mL of overnight culture were used to inoculate 250 mL of fresh LB medium (+ antibiotics). At an OD_{600} of 0.5 protein expression was induced by the addition of 1 mM IPTG for 4 h. Bacteria were harvested by centrifugation at 6,000 g for 20 min and resuspended in 25 mL PBS buffer (supplemented with Roche complete protease inhibitors). Bacteria were lysed by three cycles of French Pressing (at 14,000 psi in an HTU, Digi-F-Press, Heinemann). The resulting lysate was clarified by centrifugation at 12,000 g for 20 min. Then, the supernatant was discarded and the insoluble pellet – containing the recombinant protein – was solubilised in 10 mL 8 M urea in PBS under continuous mixing at room temperature for 2 h. The extract was passed through a 1 mL bed volume Ni-NTA Agarose (Qiagen) gravity flow

column to capture the SH2-CDR3 fusion protein via its C-terminal His-tag. To allow on-column refolding, the urea concentration was reduced over the course of several washing steps: The higher urea buffer was slowly replaced by applying 5 mL of the next lower concentration carefully on top of the Ni-NTA agarose bed and allowing complete flow-through by gravity. This procedure was repeated for a total of 5 times, thereby reducing the initial 8 M urea concentration stepwise to 4, 2, 1, 0.5 and 0.25 M. The purified and refolded fusion protein was finally eluted in 3 mL PBS buffer containing 250 mM imidazole. Samples collected during protein expression, solubilisation and purification were analysed by 12 % SDS-PAGE (see Figure S1).

5. Interaction studies of Can f 1 and SH2-CDR3 fusion peptide

96-wells microtiter plates (Nunc Maxisorb, Thermo Fisher Scientific) were coated with 100 µL of 500 µg/mL Can f 1 in coating buffer (100 mM $NaHCO_3$ pH 8.6) overnight at 4 °C. The next day, the coating solution was discarded, wells briefly washed and blocked with 1 % BSA (in coating buffer). After blocking, plates were incubated with 100 µL of 40 µg/mL SH2- CDR3 fusion protein (diluted in PBS). In the next step, 100 µL of StrepTactin-HRP conjugate (#2-1502-001, IBA Lifesciences, diluted 1:20.000 in PBS) were bound to captured fusion protein. Finally, plates were developed using 100 µL of 3, 3', 5, 5'-Tetramethylbenzidine liquid substrate (TMB, Sigma) for 30 min and measured on a Fluostar OMEGA plate reader as above. A control experiment omitting Can f 1 coating was performed to determine unspecific binding and background signal. Binding of SH2-CDR3 to Can f 1-coated wells was competed against using 10-fold molar excess of the original Can f 1-binding phage-display peptide KLWSIPTNFLLP. All experiments were performed in triplicates.
